# The Regulatory Effect and Molecular Mechanism of the Anti-Lipopolysaccharide Factor-like Gene on the Resistance of Shrimp (*Litopenaeus vannamei*) to White Spot Syndrome Virus Infection

**DOI:** 10.3390/ani15213069

**Published:** 2025-10-23

**Authors:** Bingbing Yang, Li Zhang, Fanghui Fu, Kun Luo, Sheng Luan, Jie Kong, Qiang Fu, Jiawang Cao, Baolong Chen, Ping Dai, Qun Xing, Xupeng Li, Xianhong Meng

**Affiliations:** 1 College of Fisheries and Life Science, Shanghai Ocean University, Shanghai 201306, China; nmzl19951209@163.com; 2 State Key Laboratory of Mariculture Biobreeding and Sustainable Goods, Yellow Sea Fisheries Research Institute, Chinese Academy of Fishery Sciences, Qingdao 266071, China; zhangli2780458@163.com (L.Z.); luokun@ysfri.ac.cn (K.L.); luansheng@ysfri.ac.cn (S.L.); kongjie@ysfri.ac.cn (J.K.); oucfuq@163.com (Q.F.); weifengchui555@yeah.net (J.C.); 1320040091@163.com (B.C.); daiping54@163.com (P.D.); 3 Laboratory for Marine Fisheries Science and Food Production Processes, Qingdao Marine Science and Technology Center, Qingdao 266237, China; 4 BLUP Aquabreed Co., Ltd., Weifang 261311, China; xingqun527@163.com

**Keywords:** *Litopenaeus vannamei*, *ALF-like*, White Spot Syndrome Virus, innate immunity

## Abstract

**Simple Summary:**

*Litopenaeus vannamei*, as a high-quality protein source, is widely favored by aquaculturists. However, it is frequently affected by pathogens during cultivation, among which White Spot Syndrome Virus (WSSV) is one of the most lethal pathogens in shrimp aquaculture, severely restricting the development of the shrimp industry. In this study, a resistance gene (*ALF-like*) capable of inhibiting WSSV is described. To date, no antiviral drugs developed using this gene as a target have been identified. From this perspective, the molecular mechanism study on the *ALF-like* gene in *L. vannamei* conducted herein is of great significance. This work is aimed at evaluating the potential of the *ALF-like* gene for targeted antiviral drug development. As confirmed by the study, the *ALF-like* gene can play an important anti-WSSV role in disease resistance research of *L. vannamei* and can serve as a candidate gene for developing targeted antiviral drugs.

**Abstract:**

Anti-lipopolysaccharide factors (ALFs) are an important molecular category within the antimicrobial peptide family. They play a crucial role in resisting pathogen infections and are of importance in the innate immune system of shrimp. A novel *ALF-like* gene was identified from *L. vannamei* in this study. Its expression profile was investigated after WSSV infection. Results demonstrated that the mRNA transcription level of the *ALF-like* gene was significantly upregulated in hemocytes, hepatopancreas, gills, and intestines of *L. vannamei*. When the mRNA transcription level of the *ALF-like* gene was inhibited, the expression levels of key WSSV genes (*VP 28* and *IE 1*) were significantly upregulated, accompanied by a decrease in shrimp survival rate. Meanwhile, the expression of genes involved in the apoptotic pathway (*Lv-Caspase 3*, *Lv-Caspase 8*, and *Lv-Bcl 2*) and antioxidant enzyme pathway (*Lv-GST*, *Lv-CAT*, *Lv-Prx*, *Lv-GPX*, and *Lv-SOD*) was also significantly increased. Flow cytometry further revealed that the hemocyte apoptosis rate induced by WSSV infection was reduced when the transcription level of the target gene was inhibited. These results indicate that the *Lv-ALF-like* gene plays an important regulatory role in the resistance of *L. vannamei* to WSSV infection, and studying the function of this gene is of great significance for disease prevention and control of shrimp.

## 1. Introduction

Within the category of aquatic proteins, shrimp products occupy a very important position [1,2]. However, during the breeding process, bacterial (like *Vibrio parahaemolyticus*) and viral (WSSV) infections frequently occur, resulting in substantial mortality among farmed shrimp [3,4]. White spot syndrome disease, caused by the WSSV, frequently affects shrimp and crab species [5], leading to substantial economic losses in the crustacean aquaculture industry. To date, there is no effective method to prevent the widespread transmission of this pathogen. Therefore, employing molecular biology techniques to investigate the molecular mechanisms between pathogens and their hosts is of significant importance for pathogen prevention and control, as well as for the development of healthy breeding strategies. Invertebrates lack acquired immunity and are currently believed to rely solely on their innate immune system to combat pathogens [6]. However, research on the immune system of the model organism *Drosophila* has revealed that it is primarily focused on the Toll, IMD, and JAK-STAT signaling pathways [7,8,9]. Furthermore, various immune effector molecules have been identified, including antimicrobial peptides, antibiotics, lysozyme, anti-lipopolysaccharide factors (ALFs) and diverse lectin molecules. These molecules play crucial roles in both antibacterial and antiviral immune responses [10]. Among the molecules, antimicrobial peptides (AMPs) have been crucial components of the innate immune system [11]. Notably, ALF is a specific type of AMP originally isolated from the hemolymph of *Horseshoe crabs* that exhibits activity against Gram-negative bacteria and endotoxins [12].

Antimicrobial peptides known as ALFs in crustacean aquatic animals frequently exhibit significant antibacterial activity [13,14]. Various subtypes of ALF have been identified in species such as *Penaeus vannamei*, *Penaeus monodon*, and *Fenneropenaeus chinensis* among others [15,16,17,18,19,20]. In a study on *Fenneropenaeus chinensis*, infection with WSSV led to an upward trend in *Fc-ALF* expression levels throughout the period from incubation to rapid outbreak [17]. These findings suggest that *Fc-ALF* may play a significant role in the defense against WSSV infection. Additionally, it was observed that the LBD peptides of *ALF1*, *ALF2*, *ALF5*, and *ALF7* molecules in *Penaeus chinensis* can effectively inhibit the proliferation of WSSV in in vivo application experiments [21]. The ALF molecule has also been successfully identified in studies involving *L. vannamei*, where the mRNA and protein levels of *Lv-AV-K* exhibited an increase following infection with WSSV and *Vibrio anguillarum* [22]. In the study of the molecular function of ALF in aquatic species, particularly shrimp, it was discovered that the biological role of this molecule is being progressively analyzed and revealed [23]. Following the infection of freshwater crayfish with the WSSV, the transcript level of ALF exhibited an upregulated expression trend. The application of RNA interference (RNAi) to knock down the *ALF* gene resulted in increased virus replication [24]. This study demonstrates that ALF plays a role in inhibiting the virus.

In this study, a novel *Lv-ALF-like* gene, identified in *L. vannamei*, regulates antiviral innate immunity by suppressing WSSV replication through the modulation of apoptosis and activation of antioxidant pathways. Knockdown of *Lv-ALF-like* enhances the expression of viral genes (*VP 28*, *IE 1*) and reduces shrimp survival rate; meanwhile, it inhibits the decrease in shrimp hemocyte apoptosis rate and is also involved in regulating the expression of genes related to apoptosis and antioxidant pathways. Its hemocyte-specific expression and functional divergence within the ALF family underscore its pivotal role in balancing immune responses; it plays a crucial role in pathogen prevention and control.

## 2. Materials and Methods

### 2.1. WSSV Challenge and Sample Collection

In this study, the experimental material consisted of *L. vannamei* (about 3.4 ± 0.6 g each), from Bangpu Seed Industry Technology Co., Ltd. (Weifang, China). Prior to the commencement of the experiment, a 5-day pre-incubation period was conducted, during which the salt concentration was maintained at 29‰ and the temperature at 24 ± 1 °C, and an air pump was utilized to supply oxygen, with normal feeding provided. The pathogenic WSSV, at a concentration of 4.5 × 10^6^ copies, was stored in the laboratory of the Aquatic Genetics and Breeding Center of the Yellow Sea Fisheries Research Institute of the Chinese Academy of Fishery Sciences (Qingdao, China). The immunostimulation method employed was injection, performed slowly into the abdomen of each shrimp, with 20 μL of WSSV extracted administered per shrimp. A total of 50 shrimp were used in this experiment. Among them, 40 shrimp were infected with WSSV, and 10 shrimp were used as a control group before the immunization. At specified time points 0, 12, 24, 36, 48, and 72 h post injection (hpi), the hemocytes, hepatopancreas, gills, and intestinal tissues of the shrimp were collected. Anticoagulant (10% sodium citrate, pH 7.0) was extracted using a 5 mL sterile syringe and mixed with hemolymph in equal proportions. Then, at each time point, three shrimp were sampled in parallel, and hemolymph and hemocytes were separated by centrifugation at 800× *g* for 10 min at 4 °C. Some tissues were cryopreserved, while others were utilized for total RNA extraction experiments.

### 2.2. Total RNA Extraction and cDNA Preparation

In the experiment, we utilized the RNA extraction kit supplied by Vazyme Biotechnology Co., Ltd., (Nanjing, China) to extract total RNA from shrimp tissues (50 mg tissues) following a challenge with WSSV, adhering strictly to the manufacturer’s instructions. The concentration of the extracted total RNA was determined using the NanoPhotometer^®^N50 (Implen, Schast, Germany). 10 μL of the total RNA was allocated for reverse transcription experiments, while the remaining samples were stored in a −80 °C freezer.

### 2.3. Molecular Cloning and Bioinformatic Analysis

Using the hepatopancreas of *L. vannamei* 24 h post challenge as the cDNA template, primers were designed using Primers 3.0 software (https://primer3.ut.ee/, accessed on 15 August 2024). Primers (*Lv*-ALF-F1 and *Lv*-ALF-R1) were designed to amplify the *ALF-like* gene; primer information is shown in Table 1. The experiment mainly utilized Quick Taq HS DyeMix (TOYOBO, Shanghai, China) for PCR experiments. The total reaction system was 25 μL, including 12.5 μL of 2× Quick Taq HS DyeMix (TOYOBO, Shanghai, China), 0.5 μL of each F/R primer (0.2 μM), 1 μL of cDNA template (100 ng/μL), and 10.5 μL of RNase-free ddH_2_O. The reaction program consisted of an initial denaturation at 95 °C for 3 min, followed by 35 cycles of denaturation at 95 °C for 30 s, annealing at 56 °C for 45 s, and extension at 72 °C for 50 s, concluded with a final extension at 72 °C for 5 min. The resulting products were analyzed using 1.0% agarose gel electrophoresis, purified with a purification kit, and subsequently sent to Sangon Biotech Co., Ltd. (Shanghai, China), for sequencing. The comparison of the amino acid sequence of the ALF molecule with those of other molecules is mainly analyzed using the basic local alignment search tool (BLAST 2.17.0) (http://blast.ncbi.nlm.nih.gov/Blast.cgi, accessed on 27 August 2024) on the web. The ExPASy online tool (http://www.expasy.org, accessed on 30 August 2024) was then employed to translate the gene sequences and predict the corresponding proteins. Finally, a phylogenetic tree of the proteins was constructed using the Neighbor Joining (NJ) method in MEGA 11.0 software (https://www.megasoftware.net/, accessed on 20 October 2024), with 1000 bootstrap replicates.

### 2.4. Tissue Distribution and Expression Pattern Analysis

Hemocytes, hepatopancreas, gills, intestines, and muscle tissues of healthy shrimp were collected to examine the distribution of target genes across various tissues. Concurrently, we challenged *L. vannamei* with WSSV and collected samples at different time points post challenge. The relative expression of the target genes in each tissue segment was assessed using qRT-PCR technology, following the SYBR Green kit protocol (Shanghai, China). The experimental reaction system comprised a total volume of 20 μL, including 10 μL of SYBR Green, 0.5 μL of primers, and ddH_2_O to reach the final volume. *Lv-ALF-like* and 18 S rRNA primers are listed in Table 1, and 18 S rRNA primers served as the internal reference control. The data were calculated using the 2^−ΔΔCT^ method. To enhance the stability of the experiment, three repeated trials were conducted (comprising three technical repetitions). Primer information is as shown in Table 1.

### 2.5. Double-Stranded RNA Preparation and RNA Interference

Design and synthesize specific primers, ALF and GFP, that contain the T7 promoter sequence, and follow the methodology outlined by Yang et al. [25]. Utilize the PCR-amplified product as a template for synthesizing double-stranded RNA (dsRNA), in accordance with the instructions provided in the in vitro transcription T7 kit (Takara, Japan). Prepare a total reaction volume of 50 μL, consisting of the following reagent components: 8 μg of DNA template, 20 μL of 5× transcription buffer, 2.4 μL of A/U/C/GTP mixture (10 μM each), 60 U of RNasin, and 40 U of T7 RNA polymerase. RNase-free water should be added to achieve a total volume of 50 μL. Mix the components thoroughly and incubate in a 42 °C water bath for 2 h. Subsequently, add 20 μL of 10× DNase I buffer, 6 μL of DNase I, and additional RNase-free water to reach a final volume of 100 μL. Mix well, adjust the temperature to 37 °C, and continue incubation in the water bath for 1 h. To ensure complete removal of the DNA template, purify the mixture using phenol/chloroform followed by ethanol precipitation. Prior to use, adjust the working concentration to 3 μg/μL and perform necessary interference efficiency testing before commencing formal experiments.

A total of 120 shrimp were randomly assigned to four groups, each containing 30 shrimp, and were pre-reared for 5 d during the initial phase of the experiment. During this period, the shrimp were fed normally twice a day. Subsequently, sterile 20 μL PBS and 20 μL WSSV (4.5 × 10^6^ copies) were injected into the second and third abdominal segments of the shrimp, respectively. In the interference experiment, 10 μL of dsALF RNA and dsGFP RNA (as a control group) were first injected, followed by WSSV infection. To enhance the interference effect, a booster injection was administered 24 h after the dsRNA injection. The volume of dsRNA injected was 10 μL. Statistical analysis of the interference effect and shrimp survival rate was conducted at 0, 2, 6, 12, 24, 36, 48, and 72 hpi. The experiment was designed with three parallel experiments, each consisting of three biological replicates. The internal control reference used was 18 S rRNA.

### 2.6. Detection of Immune Pathway Genes

In this experiment, 80 shrimp were randomly divided into two groups, with 40 shrimp in each group. They were fed normally twice a day. The control was dsGFP + WSSV, and the experimental group was dsALF-like + WSSV. Thirty μg of dsRNA was injected into the second and third abdominal segments of the shrimp, and then an booster injection was given 24 h later. Subsequently, 20 μL of WSSV (4.5 × 10^6^ copies) was injected for immune stimulation. Hepatopancreas tissues were collected from both groups at 0, 12, 24, 36, and 48 hpi, and total RNA was extracted for cDNA synthesis. Reverse transcribed cDNA would be used to measure relative gene expression levels.

### 2.7. Detection of Hemocytes Apoptosis Rate

To extract shrimp hemocytes from the dsGFP + WSSV and dsALF-like + WSSV groups, we mixed the hemocytes with an equal volume of anticoagulant (10% sodium citrate, pH 7.0). Subsequently, we separated the shrimp hemocytes by centrifuging at 800× *g* for 5 min at 4 °C in sterile PBS. Then, the cells were resuspended using PBS and the concentration was adjusted to 3 × 10^5^ cells/mL. For apoptosis detection, we utilized the Annexin-FITC/PI (Nanjing, China G003-1-2) apoptosis detection kit, following the manufacturer’s instructions. We combined the resuspended cells with 500 μL of binding solution, added 5 μL of Annexin-FITC, followed by 5 μL of PI (Propidium iodide), and mixed them. We incubated the mixture at room temperature in the dark for 10 min before analyzing the samples using a flow cytometer (CytoFLEX, Shanghai, China). The obtained data were analyzed using the FlowJo v10 software (https://www.flowjo.com/learn/flowjo-university/flowjo, accessed on 11 October 2024).

### 2.8. Statistical Analysis

All data in the experiment are expressed as mean ± SD (standard deviation). The data from the experiment were statistically analyzed, and then a unpaired sample *t*-test was conducted. For survival rates, we using GraphPad Prism 5.0 (https://www.graphpad.com/, accessed on 2 December 2024) software to generate the Kaplan ± Meier plot (log-rank χ^2^ test). *p* values were considered to be statistically significant as follows: * *p* < 0.05; ** *p* < 0.01; *** *p* < 0.001. The same letters indicate no significant difference, and different letters indicate significant differences between groups.

## 3. Results

### 3.1. Sequence Feature of Lv-ALF

**Figure 1:**
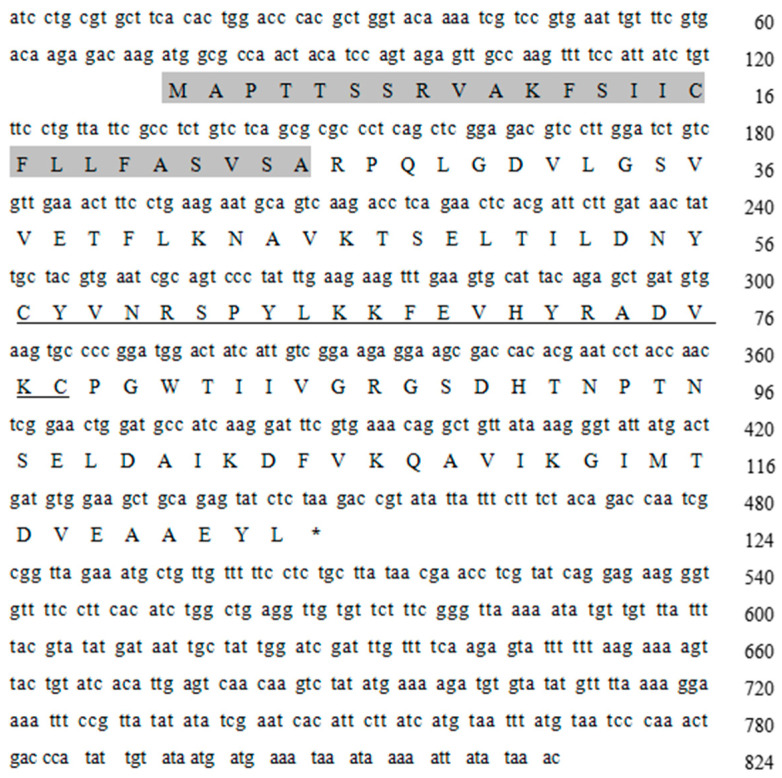
The *Lv*-*ALF-like* gene cDNA sequence and amino acid sequence. (The gray shaded region represents the signal peptide sequence, and the underlined sequence represents the LBD sequence. Uppercase letters indicate amino acids, *: Represents the termination codon).

The sequence of 824 bp was obtained through PCR amplification, resulting in an ORF of 375 bp that encodes a total of 124 amino acids. Based on an analysis using BLAST 2.17.0 software (http://blast.ncbi.nlm.nih.gov/Blast.cgi, accessed on 27 August 2024), this sequence was designated as the *Lv-ALF-like* gene. Its sequence features include a signal peptide (the gray shaded part in Figure 1), an ORF encoding 124 amino acids and a lipopolysaccharide binding domain (LBD). The LBD is indicated by an underline in Figure 1.

### 3.2. Multiple Sequence Alignment and Phylogenetic Tree

**Figure 2:**
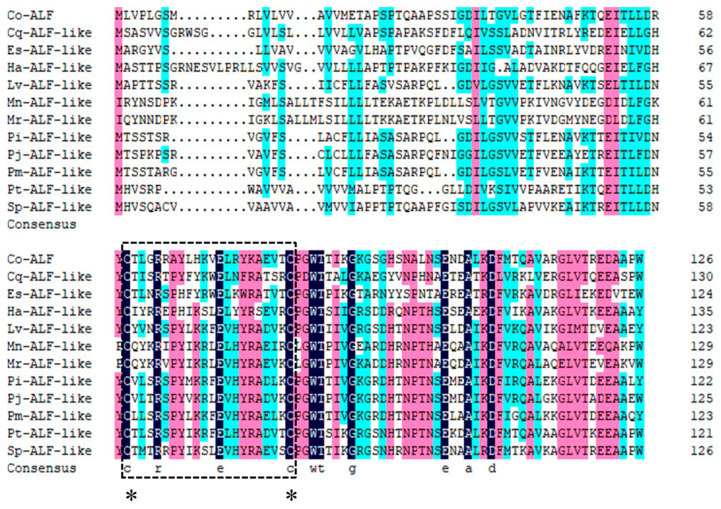
Multiple sequence alignment of ALF-like molecules. Different colors represent different conservations of amino acids (black represents fully conserved amino acid sites, red represents highly conserved amino acid sites, and blue represents moderately conserved amino acid sites, *: represents the conservative cysteine residue), and the lipopolysaccharide binding domain is marked with a square box.

To study the similarity between the *Lv*-ALF-like molecule and other molecules, we compared them with homologous sequences using DNAMAN 6.0 software (https://www.lynnon.com/, accessed on 15 August 2024), and these molecules include *Penaeus monodon* ALF-like (XP_037785278.1), *Penaeus indicus* ALF-like (XP_063590858.1), *Penaeus chinensis* ALF-like (XP_047475653.1), *Penaeus japonicus* ALF-like (XP_042866095.1), *Portunus trituberculatus* ALF-like (XP_045120962.1), *Scylla paramamosain* ALF-like (XP_063877528.1), *Chionoecetes opilio* ALF (KAG0717214.1), *Homarus americanus* ALF-like (XP_042230805.1), *Macrobrachium rosenbergii* ALF-like (XP_066959710.1), *Macrobrachium nipponense* ALF-like (XP_064081001.1), *Eriocheir sinensis* ALF-like (XP_050711929.1), and *Cherax quadricarinatus* ALF-like (XP_053652897.1). The similarity of these sequences to ALF-like molecules was found to be 53.16% (Figure 2).

**Figure 3:**
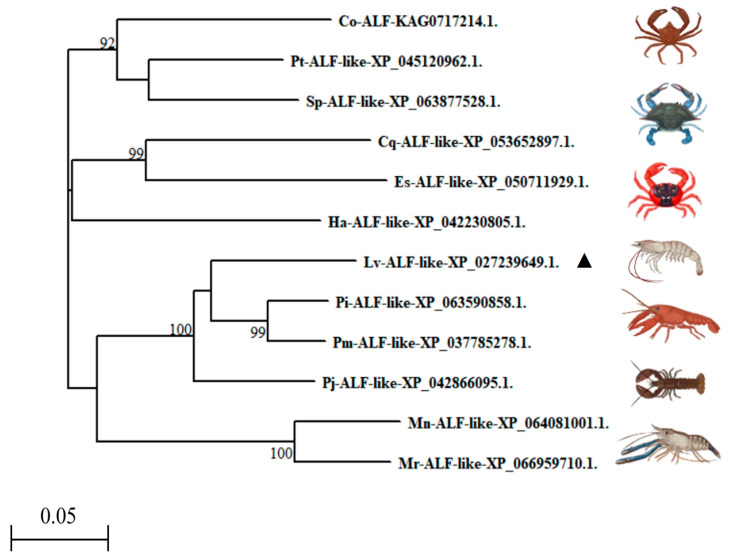
Phylogenetic tree analysis of *Lv*-ALF-like with other ALF molecules from invertebrates (▲: Representative of the *Litopenaeus vannamei*).

We further constructed a phylogenetic tree. The analysis revealed that the *Lv*-ALF-like molecule, *P. monodon* ALF-like (XP_037785278.1), *P. indicus* ALF-like (XP_063590858.1), and *P. japonicus* ALF-like (XP_042866095.1) were located on the same branch. It shared the highest similarity with *P. indicus* ALF-like (XP_063590858.1) and *P. monodon* ALF-like (XP_037785278.1). In contrast, it has been found to have lower similarity to *C. opilio* ALF (KAG0717214.1) (Figure 3).

### 3.3. Tissue Distribution and Lv-ALF like Gene mRNA Expression Pattern After WSSV Challenge

**Figure 4:**
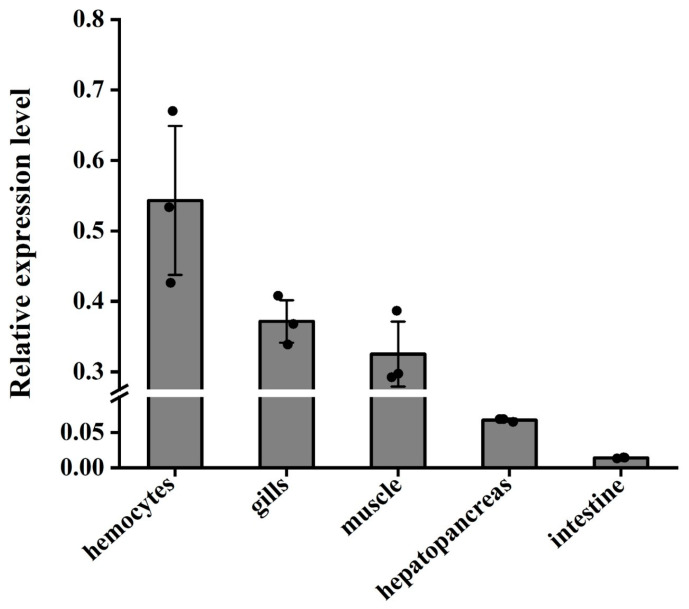
Distribution and relative expression levels of *Lv-ALF like* genes in the hemocytes, hepatopancreas, gills, intestines, and muscle tissues of normal *L. vannamei*. Tissue distribution of *ALF-like* mRNA. With 18S rRNA as the internal reference, qRT-PCR was used to detect the expression levels of ALF-like. Three independent experiments were performed, and the standard deviation was the average of three samples.

The results indicated that the *Lv-ALF like* gene exhibited the highest expression levels in hemolymph, gill, and muscle tissues, while the lowest levels were observed in the hepatopancreas and intestine, with negligible expression in intestinal tissue (Figure 4).

**Figure 5:**
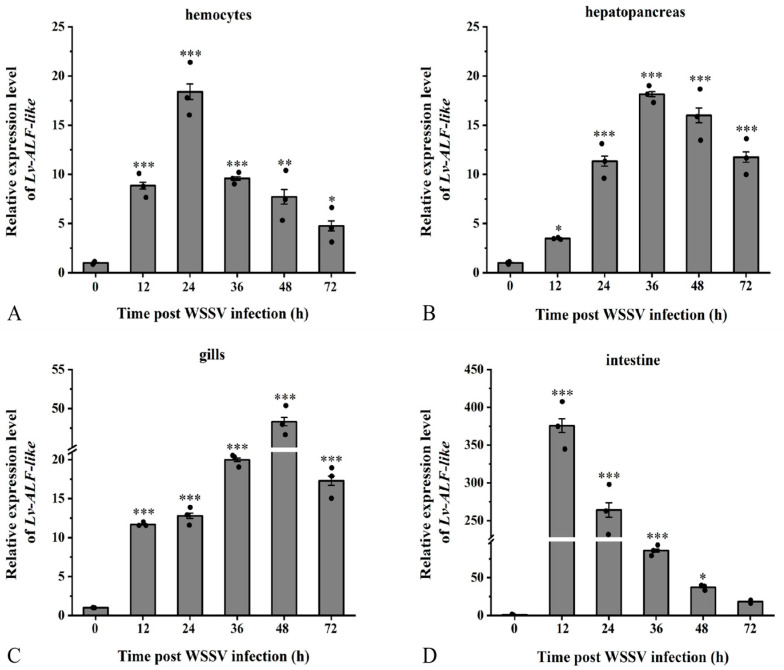
The relative expression level of the *Lv-ALF-like* gene. Study on the expression patterns in (**A**) hemocytes, (**B**) hepatopancreas, (**C**) gills, and (**D**) intestinal tissues of *L. vannamei* challenged with WSSV at different time points. Significant differences are indicated by asterisks (*: *p* <0.05, **: *p* < 0.01, ***: *p* < 0.001). Errors represent ± SD of amplification and quantitation data for three samples.

Following the challenge with WSSV injection, the mRNA expression of the *Lv-ALF like* gene in hemocytes tissue demonstrated a trend of initial increase followed by a decrease, peaking at 24 h with a relative expression level which was increased by nearly 18.5-fold (Figure 5A); gene expression in hepatopancreas tissue increased 18.2-fold at 36 h (Figure 5B). In gill tissue, gene expression peaked at 48 h, exhibiting a 48.3-fold increase (Figure 5C). Conversely, relative gene expression in intestinal tissue demonstrated an initial increase followed by a decrease, with the highest expression level occurring at 12 h, where it increased by 376-fold (Figure 5D). The expression levels of *ALF-like* in hemocytes, hepatopancreas, gills, and intestinal tissues showed an overall trend of first increasing and then decreasing at different time points after WSSV infection.

### 3.4. RNAi Interference Effect and Survival Rate Analysis

**Figure 6:**
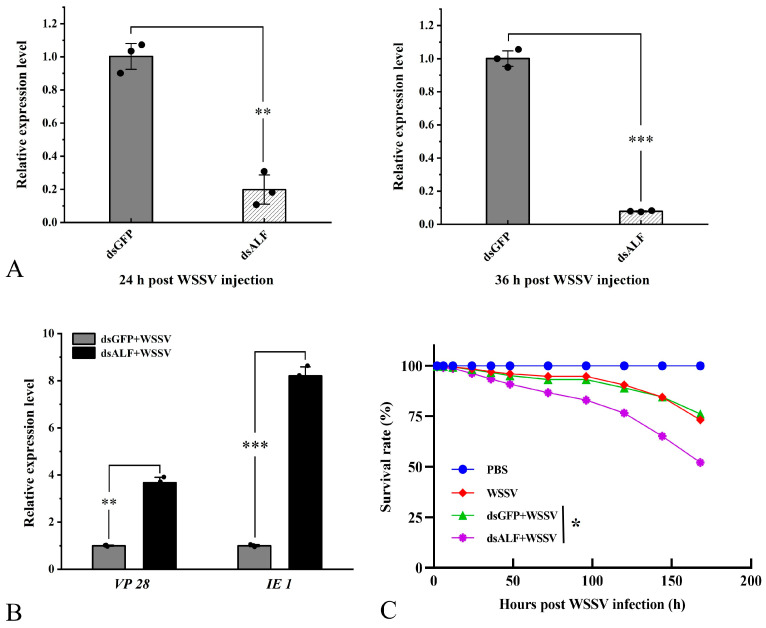
The *ALF-like* gene has the ability to inhibit viral proliferation and also affects the survival rate of shrimp. (**A**) Injection of dsALF-like RNA. qRT-PCR results show that it can significantly knock down the mRNA expression level of the target gene. (**B**) After knockdown of the *ALF-like* gene, a significant increase in the expression levels of *VP 28* and *IE 1* was observed 36 h later. (**C**) Knocking down the target gene significantly reduces the survival rate of *L. vannamei* after WSSV infection. The RNAi experiment control group was designed with dsGFP. The survival rate statistical experiment was repeated three times independently. Significant differences are indicated by asterisks (*: *p* <0.05, **: *p* < 0.01, ***: *p* < 0.001). Errors represent ± SD of amplification and quantitation data for three samples.

QRT-PCR results indicated that compared to the control injection, the expression levels of *Lv-ALF-like* were significantly reduced. It is worth noting that at 24 h and 36 h, the interference rate of *Lv-ALF-like* remained at approximately 79% and 85%, respectively (Figure 6A). In contrast, the effect of dsGFP injection on the expression levels of *Lv-ALF-like* gene was negligible.

Additionally, the proliferation of WSSV was assessed during the RNAi experiment. The results demonstrated that following the knockdown of *Lv-ALF-like*, the expression levels of *VP 28* and *IE 1* were significantly upregulated at 36 hpi (Figure 6B). Subsequently, the survival rate of shrimp in the 1 × PBS injection group, WSSV injection group, dsALF-like + WSSV injection group, and dsGFP + WSSV injection group was statistically analyzed. The experimental results indicated that the survival rate of shrimp following WSSV infection was significantly reduced after the injection of dsALF-like, compared to the other three groups (Figure 6C).

### 3.5. Lv-ALF-like Was Involved in Regulating the Antiviral Innate Immune Response

In this study, compared with the control group (dsGFP + WSSV), after *Lv-ALF-like* gene knockdown, the expression levels of apoptotic-related genes *Lv-Caspase 3* and *Lv-Caspase 8* in the experimental group (dsALF-like + WSSV) generally showed an upward trend at different time points. Compared with 12 h, their expression levels transiently decreased at 24 h, then increased again at 36 h, and gradually decreased at 48 h. This trend reflects the dynamic immune response process of “stress–compensation–reactivation–homeostasis” in *L. vannamei* after *ALF-like* knockdown. The overall upregulation represents the core activation of apoptotic clearance mechanisms, while the fluctuations at different time points may reflect the hosts’ precise temporal regulation of apoptotic pathways through negative feedback regulation, alternative pathway compensation, and damage repair mechanisms.

For the *Lv-Bax* gene, its expression in the experimental group showed an overall downward trend at 12, 24, 36, and 48 h compared with the control group. In the experimental group, the expression level decreased at 24 h compared with 12 h, recovered at 36 h, and significantly decreased at 48 h. In contrast, the *Lv-Bcl 2* gene was generally upregulated at all time points in the experimental group compared with the control group, with the highest upregulation at 12 h. These results suggest that ALF-like is not only an immune effector molecule but also participates in “immune–apoptosis crosstalk” by regulating *Bcl 2* family members. Its functional deficiency disrupts the homeostatic balance of apoptotic pathways, triggering dynamic stress responses in the host.

**Figure 7:**
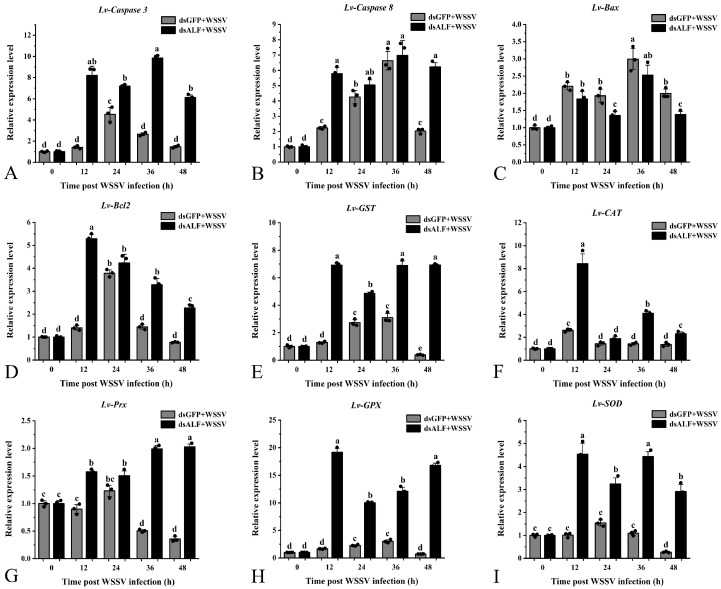
Detection of genes related to antioxidant enzyme apoptosis pathway after RNAi knockdown of *Lv-ALF like* gene. After knocking down the target gene, WSSV was injected for immune stimulation, and the expression of related genes in the shrimp hepatopancreas was detected within 0, 12, 24, 36, and 48 h later. The dsGFP + WSSV group was used as the control. The same letters indicate no differences among the groups. Different letters represent significant differences between groups. Error bars represent ± SD of three independent PCR amplifications in quantitation ((**A**) *Lv-Caspase 3*; (**B**) *Lv-Caspase 8*; (**C**) *Lv-Bax*; (**D**) *Lv-Bcl2*; (**E**) *Lv-GST*; (**F**) *Lv-CAT*; (**G**) *Lv-Prx*; (**H**) *Lv- GPX*; (**I**) *Lv-SOD*).

Following knockdown of the *Lv-ALF-like* gene, the antioxidant enzyme genes *Lv-GST*, *Lv-CAT*, *Lv-Prx*, *Lv-GPX*, and *Lv-SOD* in the experimental group were generally upregulated at 12, 24, 36, and 48 h time points compared with the control group. In the experimental group, the *Lv-GST* expression level was downregulated at 24 h compared with 12 h, then gradually recovered at 36 h and 48 h. The *Lv-CAT* expression level was highest at 12 h compared with other time points, decreased at 24 h, slightly recovered at 36 h, and dropped to the lowest level at 48 h. The *Lv-Prx* expression level continuously increased over time, reaching the highest at 48 h. The *Lv-GPX* expression level was highest at 12 h, decreased at 24 h, and then recovered at 36 h and 48 h. The *Lv-SOD* expression level was highest at 12 h, decreased at 24 h, showed a recovery trend at 36 h, and downregulated at 48 h compared with the control group (Figure 7). These results suggest that ALF-like is not only an effector molecule but also participates in host stress balance through immune–apoptosis cross-regulation. The fluctuating trend after knockdown may represent an “adaptive survival strategy” of shrimp under immune deficiency.

### 3.6. Apoptosis in Hemocytes

**Figure 8:**
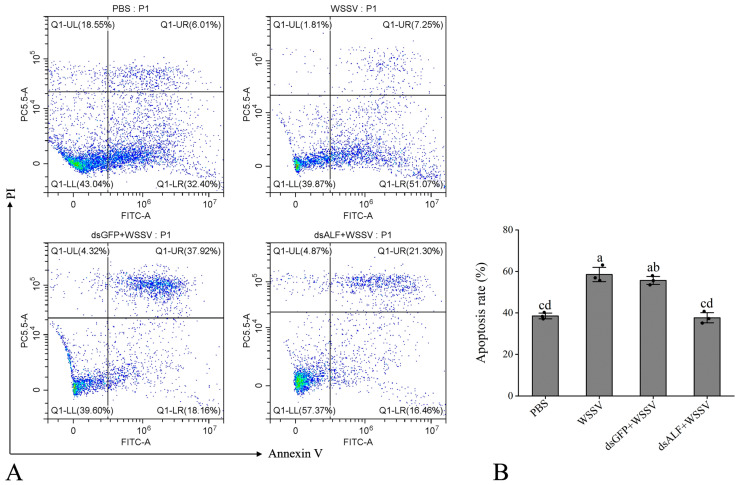
The knockdown of the *Lv-ALF-like* gene mediates the detection of the apoptosis rate in shrimp hemocytes. (**A**) Scatter plots of the experimental samples. Different quadrants in the flow cytometry analysis represent cell conditions, where Q1-UL represents cell debris; Q1-UR represents late apoptosis; Q1-LR represents early apoptosis; and Q1-LL represents normal cells. (**B**) Apoptosis rate, the total fluorescence intensity of Q1-UR and Q1-LR was used to calculate the apoptotic rate of the sample. The same letters indicate no significant difference between the groups, while different letters indicate the existence of significant differences between the groups.

The results revealed that the apoptosis rate in the PBS group was 38.41%, while in the WSSV-infected group, it was 58.32%. The dsGFP + WSSV group exhibited an apoptosis rate of 56.08%, and the dsALF-like + WSSV group showed a rate of 37.76%. Compared to the PBS group, the WSSV-infected group displayed a significant increase in apoptosis rate. In contrast, the experimental group (dsALF-like + WSSV) demonstrated a significant reduction in apoptosis rate compared to the RNAi control group (dsGFP + WSSV) (Figure 8A,B).

## 4. Discussion

Crustaceans primarily rely on innate immune responses to defend against pathogenic microorganisms [26]. Pattern recognition receptors (PRRs) in host cells identify pathogen associated molecular patterns (PAMPs), which subsequently stimulate specific signaling pathways and lead to the production of immune factors, including the AMP family [27]. Notably, research on shrimp has revealed that the ALF is a member of this antimicrobial peptide family [11]. ALF is one of the most extensively studied antimicrobial peptides in crustaceans [28] and is currently recognized as the most prevalent antibacterial factor in shrimp. While it has been demonstrated to possess both antibacterial and antiviral properties, the disease resistance mechanisms regulated by this molecule require further investigation. In our research, we identified a conserved amino acid sequence through sequence comparison, confirming that this molecule belongs to the ALF family, which we designated as the *Lv-ALF-like* gene.

Cloning and expression analysis of ALF molecules have been identified in various species, including *Pacifastacus leniusculus* [24], *Marsupenaeus japonicus* [29], and *Penaeus monodon* [13]. In this study, we analyzed the mRNA expression of *ALF-like* and found that, among the tissues examined, relative expression levels were higher in hemocyte, gills, and muscle tissues, while expression levels were either lower or not detected in the hepatopancreas and intestinal tissues. In studies involving *Penaeus monodon*, ALF was found to be constitutively expressed in hemocytes, heart, gills, intestines, and lymphoid organs, with no expression detected in the hepatopancreas [15]. This finding aligns with our results, although we observed trace expression in the hepatopancreas and intestinal tissues. Research on shrimp indicates that, despite variations in *ALF* transcript levels across different tissues, it still plays a protective role during the body’s pathogen defense processes [30].

When *L. vannamei* was stimulated by WSSV, the expression of *Lv-ALF-like* demonstrated a significant upregulation in response to pathogen stimulation. The expression profile of n*LvALF 1* in shrimp also exhibited an upregulation trend following WSSV infection [31], although variations were noted across different tissues. Previous research on AMPs has indicated that their distinct functions primarily depend on amino acid sequences [11]. Notably, when comparing the amino acid sequence of the *Lv*-ALF-like molecule with those in previous studies, it was found that they are not the same protein.

To investigate the potential role of *ALF-like* in the immune response following WSSV infection in shrimp, sequence-specific dsRNA was synthesized, leading to the successful downregulation of target gene expression via microinjection. As the *Lv-ALF-like* gene was knocked down, this resulted in the upregulation of *VP 28* and *IE 1* expression and a significant increase in cumulative mortality, which adversely affected shrimp survival rates. Through the above experiments, it was found that the *Lv-ALF-like* gene has the effect of inhibiting the proliferation of pathogens. Knockdown of the *ALF* gene led to a decrease in the survival rate of shrimp, indicating that the *Lv-ALF-like* gene plays an important inhibitory role in the shrimp’s resistance to WSSV infection. This experiment has preliminarily demonstrated that the *Lv-ALF-like* gene may play a regulatory role in the innate immune system of shrimp against viruses. In a related study by de la Vega et al., knockdown of the *Lv-ALF1* gene also led to a significant increase in shrimp mortality upon WSSV stimulation [16]. Crustins molecules belonging to the antimicrobial peptide family were observed after knocking down target gene expression by RNAi technology before injecting *vibrio* infection, and a significant increase in mortality rate in shrimp was found [32]. This is consistent with our findings. But the difference is that the specificity of the sequence may lead to them having different functions. There are similar studies on ALF antiviral in freshwater crayfish. By injecting dsRNA to interfere with the *Lv-ALF-like* gene, it was found that the WSSV content in the shrimp was significantly increased, which is consistent with the upregulated expression of *VP 28* and *IE 1* genes after knocking down the target genes [24].

In addition, after knocking down the target gene, the expression of antioxidant enzymes and apoptosis pathway genes was significantly upregulated compared with the control group dsGFP. These findings suggest that ALF are typically involved in modulating the immune response. When the expression of the *Lv-ALF-like* gene decreases, it may regulate the innate immune system by upregulating the expression of apoptotic-related signaling pathways and antioxidant enzymes, in order to enhance the organism’s defense against pathogens and maintain survival under viral infection conditions. After shrimp hemocytes were infected with WSSV, the apoptosis rate was significantly increased, indicating that WSSV infection can induce shrimp hemocyte apoptosis. After knockdown of the target gene, a significant decrease in apoptosis was also observed. It is possible that the *Lv-ALF-like* gene affects cell apoptosis by regulating the expression of the *Lv-Bcl 2* family proteins. The knockdown of *Lv-ALF-like* resulted in decreased expression of *Lv-Bax* and increased expression of *Lv-Bcl 2*, ultimately leading to a decreased hemocyte apoptosis rate. This reveals an important role of *Lv-ALF-like* in the regulation of apoptosis and provides new insights into understanding the multiple functions of *Lv-ALF-like* in the immune response. Indeed, in addition to being resistant to multiple pathogenic microorganisms, antimicrobial peptides are involved in apoptosis and promote the production of chemokines and the expression of other immune genes [33]. However, antioxidant enzyme genes (*Lv-GST*, *Lv-CAT*, *Lv-Prx*, *Lv-GPX*, *Lv-SOD*) are upregulated in response to high levels of Reactive Oxygen Species (ROS). ROS is a reactive molecule that directly kills bacteria, but excessive ROS causes tissue damage and inflammation. This could be attributed to shrimp immune deficiency caused by *Lv-ALF-like* knockdown, which leads to persistent pathogen infection, triggering ROS burst and oxidative stress, thereby driving the overall upregulation of antioxidant enzyme genes. The dynamic fluctuations of each gene reflect functional division of labor (CAT/SOD for acute response, Prx for long-term clearance, GST/GPX for metabolic detoxification), compensatory balance, and metabolic feedback, so as to maintain redox homeostasis. Upregulated expression of antioxidant enzymes (*Sp*SOD, *Sp*CAT, *Sp*GPX) can be used to remove ROS in vivo to protect the body [34]. This is consistent with our research findings. The results of the above studies show that this may be because when the expression of *ALF-like* is knocked down, the immune system may be upregulated. This upregulation is related to the expression of apoptosis-related signaling pathways and antioxidant enzymes, which serves as compensation to enhance the body’s defense against pathogens and ensure cell survival under stress conditions.

In summary, this study reveals that the *Lv*-ALF-like modulates anti-WSSV immunity in shrimp through a dual mechanism: regulating antioxidant enzyme expression to neutralize oxidative stress and controlling apoptosis-related genes to maintain cellular homeostasis. These compensatory effects enable *Lv*-*ALF-like* to exert potent anti-disease functions. The discovery of the antiviral action of *Lv-ALF-like* provides novel insights for developing targeted therapeutics and advancing preventive strategies against viral outbreaks in aquaculture.

## 5. Conclusions

This study characterized the *Lv-ALF-like* gene in *L. vannamei*; the tissue distribution analysis revealed that the expression level was highest in hemocytes and lowest in intestinal tissues. WSSV infection induced significant upregulation of *Lv-ALF-like* in immune relevant tissues (hemocytes, hepatopancreas, gills, intestines). Gene knockdown exacerbated WSSV replication, as evidenced by elevated viral gene (*VP 28*, *IE 1*) expression and reduced host survival rates, concurrently triggering upregulation of antioxidant- (*Lv-GST*, *Lv-CAT*, *Lv-Prx*, *Lv-GPX*, *Lv-SOD*) and apoptosis-related genes (*Lv-Caspase 3*, *Lv-Caspase 8*, *Lv-Bcl 2*), alongside suppressed hemocyte apoptosis. In general, the *Lv*-ALF-like protein acts as a key antiviral effector. It may play a role in inhibiting virus proliferation and enhancing host viability through the dual regulation of oxidative and apoptotic signals. However, its regulatory network remains unclear, and further mechanistic studies are needed to elucidate it.

## Figures and Tables

**Table 1:** The sequences of primers and probes used in this study.

Primer Name	Sequence (5′~3′)
Amplified sequence	
*Lv*-ALF-F	ATCGTCCGTGAATTGTTTCGT
*Lv*-ALF-R	TATGGGTCAGTTTGGGATTAC
For qRT-PCR assay	
ALF-F	TATTCGCCTCTGTCTCAGCG
ALF-R	GATGGCATCCAGTTCCGAGT
18 S-F	TATACGCTAGTGGAGCTGGAA
18 S-R	GGGGAGGTAGTGACGAAAAAT
VP 28-F	TTCTTTCACTCTTTCGGTCGT
VP 28-R	GCCAACTTCATCCTCATCAAT
IE 1-F	TGGCACAACAACAGACCCTA
IE 1-R	CTTTCCTTGAAGTACGAGAC
Caspase 3-F	AGTTAGTACAAACAGATTGGAGCG
Caspase 3-R	GGCGACAAGATGAGGCAA
Caspase 8-F	GGCGACAAGATGAGGCAA
Caspase 8-R	CAGGGTGAGGGAGAGAAAACT
Bax-F	GGTGGAATCACAAGAGAGCGA
Bax-R	TGTTCTCCACGGTGTCTCAC
Bcl 2-F	CCTTGCTTGACACAGTCGGA
Bcl 2-R	CAGACAAGGTCGTGAGGTGG
CAT-F	AGAGGGTTGTGCATGCTAAG
CAT-R	CAGCTGATCCACTCTCACCT
GST-F	TAAGGCAGGCCAAACTGTAG
GST-R	AGCTGAGGAGACCCATTCTT
Prx-F	GAAGAGCAATGCCATACGTT
Prx-R	CTTGAGCTCACGGAACTCTC
GPX-F	CCAAAGTGCATCATTTGGAC
GPX-R	CAGCAAGTTTGCGATTTCAT
SOD-F	GCGTTGGAGTGAAAGGCTCT
SOD-R	TCACGTAATCTGCACGGAGG
For dsRNA synthesis	
dsGFP-Fi	GCGTAATACGACTCACTATAGGCATCTTCTTCAAGGACGACGG
dsGFP-Ri	GCGTAATACGACTCACTATAGGAGTTCACCTTGATGCCGTTCT
*Lv*-ALF-Fi	GCGTAATACGACTCACTATAGGCGTCCTTGGATCTGTCGTTG
*Lv*-ALF-Ri	GCGTAATACGACTCACTATAGGATCCAGTTCCGAGTTGGTAGG

## Data Availability

The original contributions presented in the study are included in the article; further inquiries can be directed to the corresponding authors.
